# Curvilinear association between Framingham Steatosis Index and chronic kidney disease: a nationwide cross-sectional study

**DOI:** 10.3389/fmed.2024.1518202

**Published:** 2025-01-14

**Authors:** Chunqi Jiang, Bo Wang, Jun Wang, Yinuo Qu, Xin Zhang

**Affiliations:** ^1^Affiliated Hospital of Shandong University of Traditional Chinese Medicine, Jinan, Shandong, China; ^2^Central Hospital of Jinan City, Jinan, Shandong, China; ^3^College of Acupuncture - Moxibustion, Shandong University of Traditional Chinese Medicine, Jinan, Shandong, China

**Keywords:** Framingham Steatosis Index, chronic kidney disease, curvilinear, NHANES, cross-sectional study

## Abstract

**Introduction:**

Fatty liver disease is potentially linked to chronic kidney disease (CKD), yet the association between the Framingham Steatosis Index (FSI) and CKD remains uncharted. Our study thoroughly investigated the correlation between FSI and CKD, aiming to elucidate the underlying links between these two conditions.

**Methods:**

The relationship between FSI and CKD was evaluated using a weighted multivariate logistic regression model, and the curvilinear relationship between FSI and CKD was explored through smooth curve fitting. We engaged a recursive partitioning algorithm in conjunction with a two-stage linear regression model to determine the inflection point. By conducting stratified analyses, the heterogeneity within subpopulations was explored.

**Results:**

In the fully adjusted Model 3, which accounted for all covariates, the odds ratios (ORs) (95% CI) for the association between FSI and CKD were 1.01 (0.97, 1.06), indicating no significant statistical association. Sensitivity analysis confirms the stability of the relationship between FSI and CKD. Smooth curve fitting discloses a non-linear association between FSI and CKD. The two-piecewise linear regression model, applied to explore this non-linearity, identified an inflection point at an FSI value of −3.21. Below this threshold, the OR (95% CI) was 0.25 (0.17, 0.37), signifying an inverse correlation between FSI and CKD. Above the inflection point, the OR (95% CI) was 1.19 (1.13, 1.25), suggesting a positive correlation. In the stratified curve analysis, the results were essentially consistent with the overall findings, except for the subgroups with BMI > 30 and age > 50.

**Conclusion:**

We found a curvilinear relationship between FSI and CKD.

## 1 Introduction

Chronic kidney disease (CKD) stands as a significant challenge to global health, with approximately 700 million people worldwide currently affected, and this figure is continuing to grow (1). Predictive models suggest that CKD could emerge as the fifth leading cause of mortality by 2040 (2). Beyond its high incidence, CKD engenders a spectrum of severe health issues and outcomes. Research has firmly established links between CKD and conditions such as frailty, cardiovascular complications, and renal failure (3, 4). Furthermore, recent studies have highlighted associations between CKD and various metabolic markers, including residual cholesterol, the waist triglyceride index, and the triglyceride-glucose index (5–7).

The Framingham Steatosis Index (FSI) emerged from a comprehensive cross-sectional study conducted by Long MT and colleagues in 2016, which involved 1,181 subjects. Through a meticulous stepwise regression analysis, the study identified demographic and clinical factors, as well as biochemical markers associated with hepatic steatosis (8). This index stands as a economical, and efficient tool for the detection of individuals with hepatic steatosis. In clinical studies, FSI has demonstrated remarkable effectiveness. Research has confirmed that the FSI serves as a dependable predictive marker for non-alcoholic fatty liver disease (NAFLD) (9). Moreover, the FSI has exhibited its efficacy in identifying metabolic associated fatty liver disease (MAFLD) within Eastern populations (10). In addition, a cohort study has highlighted a correlation between the FSI and the risks of cardiovascular (CV) diseases and mortality (11).

Current literature demonstrates a significant association between steatohepatitis (SLD) and CKD, with advanced liver fibrosis being linked to CKD (12). Both MAFLD and alcoholic liver disease (ALD) have been shown to have independent associations with the development of CKD (13). Additionally, the fatty liver index (FLI), another biomarker for fatty liver disease, has been validated for identifying individuals at high risk for CKD events (14). CKD has been correlated with hypertension and diabetes (15). However, the relationship between FSI—a robust surrogate marker for hepatic steatosis that incorporates hypertension and diabetes—and CKD remains unexplored. Our objective is to leverage data from the U.S. National Health and Nutrition Examination Survey (NHANES) spanning from 2005 to 2020 for a cross-sectional analysis to clarify the connection between FSI and CKD. This study aims to evaluate the potential of FSI as a predictive indicator for the risk of CKD events.

## 2 Materials and methods

### 2.1 Study participants

**FIGURE 1:**
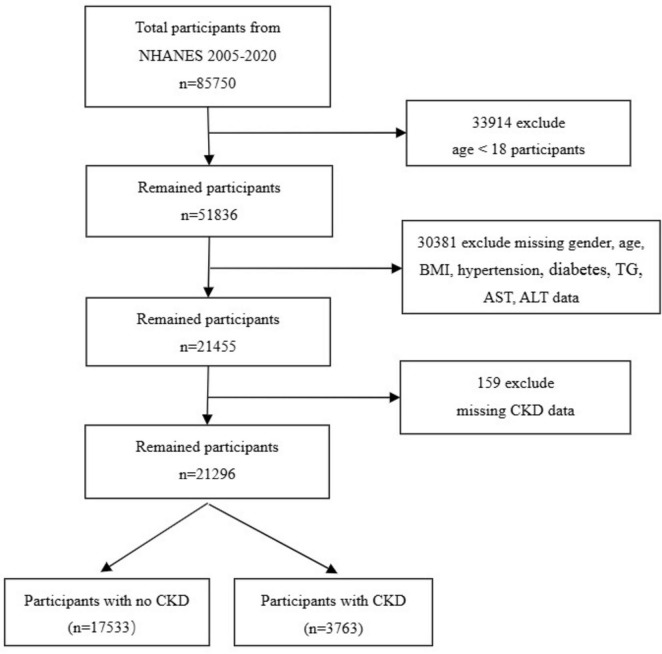
Flow chart of sample selection from the 2005–2020.

Our research utilizes data from the U.S. NHANES, covering eight survey cycles from 2005 to 2020. It includes a wide array of information such as demographics, lifestyle habits, physical measurements, and blood biochemistry, all of which were collected via in-home interviews, mobile examination centers (MECs), and lab tests. This database is publicly accessible, requiring no special permissions for researchers to utilize the data. The National Center for Health Statistics Research Ethics Review Board granted approval for the study protocol. Consequently, all participants offered their written informed consent. To ensure participant privacy, all personally identifiable information was anonymized. During data processing, we excluded 33,914 subjects under the age of 18, 30,381 participants with missing data on gender, age, hypertension, diabetes, triglycerides (TG), Aspartate aminotransferase (AST), alanine aminotransferase (ALT), and BMI, and 159 individuals lacking CKD data, resulting in a final study cohort of 21,296 subjects (Figure 1).

### 2.2 Study variables

#### 2.2.1 Definition of CKD

CKD definition adheres to the guidelines provided by the Kidney Disease: Improving Global Outcomes (KDIGO) Glomerular Diseases Work Group. A diagnosis of CKD is warranted with the fulfillment of one or more of the following conditions for a period of 3 months or more: (1) an estimated glomerular filtration rate (eGFR) that is persistently below 60 mL/min/1.73 m2, as determined by the CKD Epidemiology Collaboration (CKD-EPI) creatinine equation. (2) A urine albumin-to-creatinine ratio (UACR) that surpasses the threshold of 30 mg/g (16).

#### 2.2.2 Definition of FSI

Our research is anchored on the formula meticulously derived by Long MT and collaborators in their 2016 study (8). The FSI was determined using the formula: FSI = −7.981 + 0.011 × age (years)-0.146 × sex (female = 1, male = 0) + 0.173 × BMI (kg/m^2^) + 0.007 × triglycerides(mg/dl) + 0.593 × hypertension (yes = 1, no = 0) + 0.789 × diabetes (yes = 1, no = 0) + 1.1 × ALT/AST ratio ≥ 1.33(yes = 1, no = 0). AST, ALT and TG were meticulously assessed by specialists, who were trained in accordance with the rigorous standards set by the National Center for Health Statistics (NCHS) and executed under the guidance of the Centers for Disease Control and Prevention (CDC) protocols. The blood lipid levels were measured from peripheral blood samples collected in the morning after at least 8 h of fasting. Enzymatic methods were used to determine the serum levels of TG and AST. Determine serum or plasma ALT levels employing a kinetic rate assay.

#### 2.2.3 Assessment of other variables

CDC meticulously gathered comprehensive participant data through computer-assisted personal interviews, covering demographics, lifestyle practices, self-reported health statuses, physical dimensions, and biochemical indices. Our analysis meticulously scrutinized key demographic factors, such as age, gender, ethnicity, educational attainment, marital status, and the income-to-poverty ratio. Lifestyle elements, including smoking and drinking habits as well as recreational activities, were also considered. Self-reported health data concentrated on prevalent conditions like diabetes, hypertension, and cardiovascular disease, with a particular focus on BMI for anthropometric assessment. Biochemical data assessing liver, kidney, and metabolic health, including gamma-glutamyl transferase (GGT), TG, high-density lipoprotein cholesterol (HDL-C), low-density lipoprotein cholesterol (LDL-C), serum uric acid (SUA), blood urea nitrogen (BUN), ALT, AST, serum creatinine (SCR), and urinary albumin (UA), with the latter being collected directly from the participants on-site. Smoking status was classified into three distinct categories: “Never” was defined as having smoked fewer than 100 cigarettes in one’s lifetime; “Former” referred to those with a previous smoking history who had since quit; and “Now” was designated for individuals who continued to smoke (17). Participation in recreational activities was binary, recorded as either “Yes” or “No.” Diabetes (including pre-diabetes) was diagnosed based on meeting any of the following criteria: (1) fasting blood glucose levels above 7.0 mmol/L; (2) glycosylated hemoglobin (HbA1c) levels at or exceeding 6.5%; (3) random blood glucose levels of at least 11.1 mmol/L; (4) 2-h oral glucose tolerance test (OGTT) glucose levels reaching or surpassing 11.1 mmol/L; (5) a formal diagnosis of diabetes by a healthcare provider; (6) impaired fasting glucose (IFG) ranging from 6.11 to 7.0 mmol/L or impaired glucose tolerance (IGT) with OGTT levels between 7.7 and 11.1 mmol/L. Hypertension was identified through one or more of the following conditions: (1) systolic blood pressure readings of 140 mmHg or higher; (2) diastolic blood pressure readings of 90 mmHg or higher; (3) the current use of antihypertensive medications; (4) self-reported hypertension. Alcohol consumption levels were categorized as follows: “Heavy” drinking was characterized for women as three or more drinks daily or four or more drinks on a single occasion, and for men as four or more drinks daily or five or more on a single occasion, with binge drinking occurring on at least 5 days per month. “Moderate” drinking was defined as two drinks daily for women and three for men, with binge drinking happening on at least 2 days per month. “Mild” drinking involved one drink per day for women and two for men. “Never” drinking was applied to those who had fewer than 12 drinks in their lifetime, while “Former” drinkers were individuals with a history of alcohol consumption but who no longer drank. Cardiovascular disease (CVD) was ascertained using a medical history questionnaire that recorded whether participants had been diagnosed by a physician with conditions such as coronary artery disease, congestive heart failure, or had a history of heart attack (18).

### 2.3 Statistical analysis

The study data were appropriately weighted to accurately reflect the broader demographic. Participants were classified into groups with or without CKD, based on their baseline characteristics. In presenting the data, continuous variables including age and BMI were reported with means and standard errors, whereas categorical variables such as gender and smoking status were presented as percentages of the overall sample. In order to investigate the relationship between FSI and CKD, we utilized weighted logistic regression analyses. The results were expressed in terms of odds ratios (ORs) and their respective 95% confidence intervals (95% CIs), providing a measure of association and precision. To affirm the reliability of the relationship between FSI and CKD, we performed a linear trend test and carried out sensitivity analyses. Furthermore, to examine the potential for a non-linear association between FSI and CKD, we conducted a restricted cubic spline analysis. Ultimately, to identify the inflection point of curvilinear, we employed a recursive algorithm coupled with a two-stage linear regression model. Stratified analysis was employed to assess the presence of specific populations.

## 3 Results

### 3.1 Baseline characteristics

Table 1 delineates the demographic and clinical characteristics of the study participants. Notably, those with CKD exhibit significantly higher levels of age, BMI, TG, SUA, SCR, BUN, UA, GGT, and FSI. Conversely, they display lower income to poverty ratios, educational attainment and LDL-C. Within CKD patient population, there is a notably higher proportion of women, as well as individuals who are divorced, widowed, or separated, and those who do not engage in leisure activities. Furthermore, a predisposition toward CVD, diabetes, and hypertension is more pronounced among participants with CKD.

**TABLE 1:** Baseline characteristics of participants.

	No CKD (*n* = 17533)	With CKD (*n* = 3763)	*P*-value
Age (year)	44.96 ± 16.18	60.37 ± 17.69	<0.0001
Sex (%)			<0.0001
Female	49.80	57.26	
Male	50.20	42.74	
Race/ethnicity (%)			0.0009
Mexican American	8.79	8.29	
Non-hispanic white	66.71	67.11	
Non-hispanic black	10.40	12.48	
Other hispanic	6.01	4.94	
Other race	8.10	7.18	
Marry status (%)			<0.0001
Never married	18.73	10.30	
Married/Living with partner	65.00	58.08	
Divorced/Widowed/ Separated	16.27	31.61	
Education status (%)			<0.0001
Less than high school	4.79	9.20	
High school	33.73	41.03	
More than high school	61.48	49.77	
Drinking status (%)			<0.0001
Never	10.05	15.70	
Mild	38.20	36.99	
Moderate	18.76	13.17	
Heavy	22.57	13.93	
Former	10.42	20.21	
Recreational activity (%)			<0.0001
No	43.83	59.31	
Yes	56.17	40.69	
Smoking status (%)			<0.0001
Never	55.61	51.95	
Now	20.04	16.84	
Former	24.35	31.21	
CVD (%)			<0.0001
Yes	6.58	25.75	
No	93.42	74.25	
Diabetes (%)			<0.0001
Yes	28.55	59.12	
No	71.45	40.88	
Hypertension (%)			<0.0001
Yes	31.69	67.00	
No	68.31	33.00	
Income to poverty ratio	3.05 ± 1.64	2.66 ± 1.59	<0.0001
BMI (kg/m2)	28.79 ± 6.79	30.30 ± 7.89	<0.0001
TG(mmol/L)	1.35 ± 1.10	1.59 ± 1.29	<0.0001
FSI	−1.40 ± 1.71	−0.68 ± 1.87	<0.0001
HDL-C (mmol/L)	1.40 ± 0.41	1.40 ± 0.48	0.5393
LDL-C (mmol/L)	2.94 ± 0.90	2.77 ± 0.98	<0.0001
SUA (μmol/L)	320.57 ± 78.69	356.84 ± 99.08	<0.0001
SCR (μmol/L)	74.53 ± 15.11	98.80 ± 76.50	<0.0001
BUN (mmol/L)	4.62 ± 1.45	6.38 ± 3.29	<0.0001
UA(mg/L)	9.66 ± 9.64	203.80 ± 779.29	<0.0001
GGT (U/l)	26.76 ± 32.41	35.04 ± 66.69	<0.0001
AST (U/l)	24.79 ± 20.22	23.01 ± 16.64	<0.001
ALT (U/l)	24.64 ± 19.33	25.17 ± 16.33	0.032

P (continuous variables): calculated by weighted linear regression model. P (categorical variables): calculated by weighted chi-square test.

### 3.2 Association of FSI and CKD

As shown in Table 2, in the unadjusted Model 1, the ORs for FSI were 1.26 (95% CI 1.23, 1.28), indicating a 26% increased risk for CKD with each unit rise in FSI. Following adjustment for age, sex, and ethnicity in Model 2, the ORs reduced to 1.20 (95% CI 1.18, 1.23), corresponding to a 20% increased risk of CKD per unit increase in FSI. Upon further comprehensive adjustment in Model 3, which included variables such as educational attainment, poverty income ratio, marital status, smoking behavior, recreational activities, LDL-C, HDL-C, SUA, SCR, BUN, and the presence of diabetes, hypertension, and CVD, the ORs were attenuated to 1.01 (95% CI 0.97, 1.06), suggesting no significant link between FSI and CKD risk.

**TABLE 2:** Association of FSI and CKD.

Exposure	Model 1 OR (95% CI)	*P-*value	Model 2 OR (95% CI)	*P*-value	Model 3 OR (95% CI)	*P*-value
FSI	1.26 (1.23, 1.28)	<0.0001	1.20 (1.18, 1.23)	<0.0001	1.01 (0.97, 1.06)	0.5874
**FSI quartile**						
Q1	Reference		Reference		Reference	
Q2	2.05 (1.81, 2.31)	<0.0001	1.02 (0.90, 1.17)	0.7229	0.82 (0.68, 0.99)	0.0366
Q3	2.82 (2.51, 3.17)	<0.0001	1.25 (1.10, 1.42)	0.0008	0.76 (0.62, 0.93)	0.0070
Q4	3.66 (3.27, 4.11)	<0.0001	2.01 (1.77, 2.27)	<0.0001	0.87 (0.69, 1.09)	0.2284
P for trend	<0.0001		<0.0001		0.4649	
**Sex**						
Female	1.25 (1.22, 1.28)	<0.0001	1.15 (1.11, 1.18)	<0.0001	0.94 (0.88, 1.00)	0.0458
Male	1.27 (1.23, 1.31)	<0.0001	1.29 (1.25, 1.34)	<0.0001	1.14 (1.05, 1.23)	0.0009
**BMI**						
≤30	1.37 (1.32, 1.43)	<0.0001	1.09 (1.04, 1.14)	0.0004	0.73 (0.64, 0.83)	<0.0001
>30	1.28 (1.24, 1.33)	<0.0001	1.31 (1.26, 1.37)	<0.0001	1.11 (1.03, 1.19)	0.0074
**Age**						
≤50	1.21 (1.18, 1.25)	<0.0001	1.23 (1.19, 1.28)	<0.0001	1.00 (0.93, 1.08)	0.9432
>50	1.20 (1.16, 1.23)	<0.0001	1.20 (1.17, 1.24)	<0.0001	0.98 (0.92, 1.04)	0.4987

Model 1: no adjustment. Model 2: adjusted for age, gender, and race. Model 3: adjusted for gender, age, race, hypertension, family income to poverty ratio, education level, smoking status, marriage status, drinking status, physical activity, LDL-C, HDL-C, SUA, SCR, BUN, diabetes, and CVD. In the subgroup analyses, which are stratified by sex, BMI, or age, the model does not incorporate adjustments for the stratification variables themselves.

Sensitivity analyses, using Q1 as the reference, showed ORs (95% CI) of 0.82 (0.68, 0.99) for Q2, 0.76 (0.62, 0.93) for Q3, and 0.87 (0.69, 1.09) for Q4. These results hint at a possible non-linear relationship between FSI levels and the development of CKD.

When stratified by sex, a significant positive association between FSI and CKD was observed among males across all models, with an OR (95% CI) of 1.14 (1.05, 1.23) in the fully adjusted Model 3. However, for females, the association was significantly inverse in Model 3, with an OR (95% CI) of 0.94 (0.88, 1.00). BMI-stratified analysis revealed a significant inverse correlation between FSI and CKD in individuals with a BMI ≤ 30 in the fully adjusted Model 3, while an opposite, significant positive correlation was noted in those with a BMI > 30. Age-stratified analysis demonstrated no significant correlation between FSI and CKD across all age groups in the fully adjusted Model 3.

**FIGURE 2:**
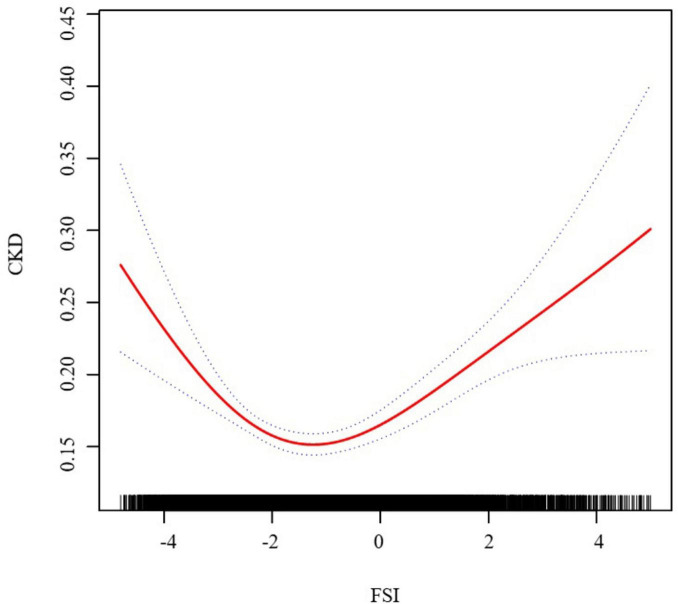
The association between FSI and CKD. We adjusted for gender, age, race, family income to poverty ratio, education level, smoking status, marry status, drinking status, physical activity, LDL-C, HDL-C, SUA, SCR, BUN, diabetes, and CVD.

**FIGURE 3:**
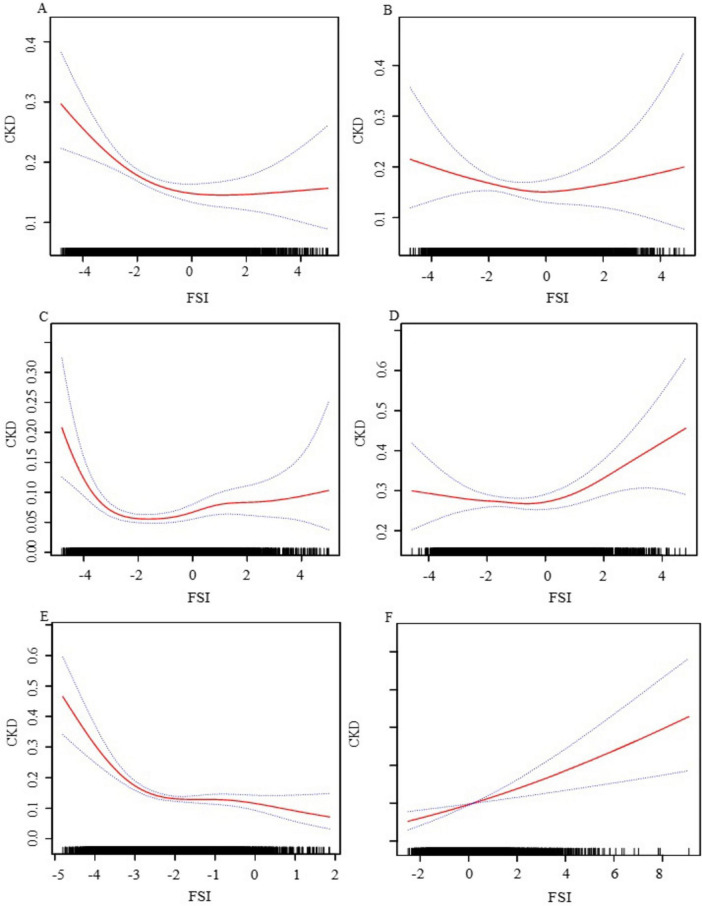
The association between FSI and CKD stratified by gender, age, and BMI. **(A)** Female; **(B)** male; **(C)** age ≤ 50; **(D)** age > 50; **(E)** BMI ≤ 30; **(F)** BMI > 30. We adjusted for gender, age, race, family income to poverty ratio, education level, smoking status, marry status, drinking status, physical activity, LDL-C, HDL-C, SUA, SCR, BUN, diabetes, CVD and hypertension. In subgroup analyses, the model is not adjusted for the stratification variables themselves.

We have delved into an in-depth analysis to elucidate the non-linear relationship between FSI and CKD, rigorously controlling for all covariates. As depicted in Figure 2, the smooth curve we have plotted substantiates the presence of a non-linear association between FSI and CKD. Utilizing a two-piecewise linear regression model, a key inflection point was discerned at −3.21. Below this threshold, the OR (95% CI) of 0.25 (0.17, 0.37) signifies a notably inverse correlation. Conversely, above the inflection point, the OR (95% CI) of 1.19 (1.13, 1.25) suggests a significant positive correlation, further elaborated in Table 3. In the stratified analysis, we noted significant differences in the age and BMI subgroups, whereas other results were largely consistent (Figure 3). Within the BMI stratification, participants with a BMI greater than 30 exhibited a linear positive correlation between FSI and CKD. In contrast, those with a BMI of 30 or lower showed a curvilinear relationship, which was in line with the overall findings. For participants over the age of 50, the curvilinear relationship between FSI and CKD had an inflection point at −1.52. Prior to this inflection point, the OR with its 95% CI was 1.39 (1.17, 1.66), reflecting a significant positive correlation. Post-inflection, the OR (95% CI) was 1.06 (0.98, 1.16), indicating no significant correlation. Notably, the inflection point for this cohort was markedly lower compared to other groups.

**TABLE 3:** Threshold effect analysis of FSI on CKD using a two-piecewise linear regression model.

Outcome:	Inflection point	OR (95% CI)	*P-*value
Total	<−3.21	0.25 (0.17, 0.37)	<0.0001
	>−3.21	1.19 (1.13, 1.25)	<0.0001
**Sex**			
Female	<−3.25	0.36 (0.23, 0.58)	<0.0001
	>−3.25	1.07 (1.00, 1.15)	0.0518
Male	<−3.31	0.14 (0.06, 0.37)	<0.0001
	>−3.31	1.35 (1.26, 1.46)	<0.0001
**Age**			
≤50	<−3.16	0.31 (0.21, 0.46)	<0.0001
	>−3.16	1.30 (1.20, 1.42)	<0.0001
>50	<−1.52	1.39 (1.17, 1.66)	0.0002
	>−1.52	1.06 (0.98, 1.16)	0.1617
**BMI**			
≤30	<−3.28	0.22 (0.14, 0.34)	<0.0001
	>−3.28	1.13 (1.03, 1.25)	0.0112

We adjusted for gender, age, race, family income to poverty ratio, education level, smoking status, marry status, drinking status, physical activity, LDL-C, HDL-C, SUA, SCR, BUN, diabetes, and CVD.

## 4 Discussion

Based on our comprehensive knowledge, this is the inaugural large-sample study to investigate the association between FSI and the likelihood of CKD. Our analysis, after meticulously accounting for all potential confounding factors, uncovered a non-linear association between FSI and CKD. The trend revealing the inverse relationship between FSI and CKD at lower values of FSI, followed by a positive correlation at higher values, was pronounced and statistically significant. Utilizing a two-piecewise linear regression model, we pinpointed an inflection point at an FSI score of −3.21. Below this threshold, the odds ratio (OR) (95% CI) was 0.25 (0.17, 0.37), indicating a significant inverse correlation. Above the inflection point, the risk of CKD increased by 19% for each unit rise in FSI, with an OR (95% CI) of 1.19 (1.13, 1.25). The distinct association observed before and after the inflection point is of significant statistical consequence, underscoring the intricate interplay between FSI and CKD.

The computation of FSI is intricate, incorporating demographic details, biochemical markers, and clinical parameters, including age, sex, BMI, triglyceride levels, hypertension, diabetes, ALT, and AST. To date, the relationship between FSI and CKD in a clinical context has not been explored. Nevertheless, studies examining the links between these individual elements and CKD have demonstrated considerable advancement. Research has demonstrated that increased ALT levels are linked to CKD, and a reduced AST/ALT ratio is associated with CKD (19). With the progression of CKD, there is a significant reduction in serum transaminase levels (20). The study by Qin S et al., utilizing multivariate analysis, established age and diabetes as independent risk factors for CKD (21). Furthermore, a meta-analysis conducted by Ardavani et al. corroborated that diabetes and hypertension elevate the risk of CKD (22).

While the precise pathophysiological mechanisms linking CKD development to FSI remain unclear, several possibilities have been postulated. Inflammation, glucose metabolism dysregulation, and lipid metabolism disturbances are considered crucial in the interplay between FSI and CKD.

Hypertension, obesity, elevated ALT/AST ratios, and TG can initiate inflammatory responses that result in damage to both glomeruli and renal tubules. With the advancement of CKD, there is a notable decline in serum transaminase levels, potentially associated with vitamin B6 deficiency, the accumulation of lactic acid and uremic toxins, the induction of hepatocyte growth factor, and fluid retention (23). Hypertension may stimulate endothelial cell (EC) activation and subsequent dysfunction, precipitating inflammatory reactions (24). Obesity activates insulin resistance and pro-inflammatory signaling pathways, thereby triggering inflammation (25). An increased ALT/AST ratio is indicative of insulin resistance and acts as a biomarker for systemic inflammation and oxidative stress (26). The cytotoxic effects of TG can exacerbate lipid accumulation within cells. Fat-derived factors, including leptin, tumor necrosis factor-alpha (TNF-α), and angiotensin II, promote the advancement of oxidative stress and inflammation. These inflammatory responses ultimately culminate in damage to the renal glomeruli and tubules (27). Insulin resistance, induced by the aforementioned factors, can activate the sympathetic nervous system, promote sodium retention, and downregulate the natriuretic peptide system, culminating in impaired renal physiology and the progression of CKD (28). Conversely, the decline in renal function may exacerbate insulin resistance (29), resulting in hyperglycemia, hypertriglyceridemia, and hypertension (30). Furthermore, CKD can impair the metabolism of very-low-density lipoprotein (VLDL), reduce the activity of lipoprotein lipase (LPL), and consequently elevate TG levels (31).

Research indicates that the TyG-BMI index, encompassing BMI, TG, and glucose levels, correlates more robustly with the incidence of CKD in female. In contrast, another investigation revealed that, the disease progression is more rapid in male CKD patients, a disparity potentially attributable to the effects of sex hormones (32, 33). Additionally, significant variations in inflammation, oxidative stress, and insulin resistance are observed across different genders and age groups (34, 35). These factors could plausibly elucidate the observed disparities.

Our data is sourced from the NHANES database, which is well-regarded for its stringent data collection protocols and extensive sampling, thereby lending substantial credibility and reliability to our research findings. By employing various analytical methods, including multiple regression equations, stratified analyses, threshold effects, and curve fitting, we have thoroughly examined the connection between FSI and CKD, meticulously exploring how this relationship manifests differently among various demographic groups. However, our study has its inherent limitations. Firstly, as a cross-sectional observational study, it does not confirm a causal relationship between FSI and CKD, highlighting the need for longitudinal studies to determine causality and the chronological order of events. Secondly, despite considering a comprehensive array of covariates, there may still be unmeasured confounding factors that could influence the relationship between FSI and CKD, such as individual lifestyle choices and genetic propensities. Thirdly, variations in socioeconomic status and access to healthcare may also affect the research outcomes. Through concerted efforts, our objective is to elucidate the potential mechanisms underlying the FSI and CKD association.

## 5 Conclusion

By leveraging NHANES data from 2005 to 2020, this study has identified a non-linear association between FSI and CKD, with a critical inflection point at an FSI value of −3.21. This complex interplay emphasizes the pivotal role of demographic and clinical factors in the assessment of CKD risk. Given the intricacy of FSI, a standardized approach to CKD management may not suffice. Instead, there is an accentuated need for individualized interventions that include lifestyle adjustments, chronic disease management, and lipid profile modulation. Moreover, the observed variations in the FSI-CKD association across different demographic groups highlight the imperative for customized management strategies. These interventions are crucial not only for preventing or decelerating CKD progression but also for elevating the overall quality of care for at-risk individuals, with the ultimate goal of enhancing patient outcomes across various populations.

## Data Availability

Publicly available datasets were analyzed in this study. This data can be found here: www.cdc.gov/nchs/nhanes/Default.aspx.

## References

[1] GBD 2019 Diseases and Injuries Collaborators. Global, regional, and national burden of chronic kidney disease, 1990–2017: a systematic analysis for the Global Burden of Disease Study 2017. *Lancet (London, England).* (2020) 395:709–33. 10.1016/S0140-6736(20)30045-3 32061315 PMC7049905

[2] Foreman KJ Marquez N Dolgert A Fukutaki K Fullman N McGaughey M Forecasting life expectancy, years of life lost, and all-cause and cause-specific mortality for 250 causes of death: reference and alternative scenarios for 2016-40 for 195 countries and territories. *Lancet.* (2018) 392:2052–90. 10.1016/s0140-6736(18)31694-5 30340847 PMC6227505

[3] Zheng G Cheng Y Wang C Wang B Zou X Zhou J Elucidating the causal nexus and immune mediation between frailty and chronic kidney disease: integrative multi-omics analysis. *Ren Fail.* (2024) 46:2367028. 10.1080/0886022X.2024.2367028 39010723 PMC11265307

[4] Cockwell P Fisher LA. The global burden of chronic kidney disease. *Lancet (London, England).* (2020) 395:662–4. 10.1016/S0140-6736(19)32977-0 32061314

[5] Yuan Y Hu X Zhang S Wang W Yu B Zhou Y Remnant cholesterol, preinflammatory state and chronic kidney disease: association and mediation analyses. *Ren Fail.* (2024) 46:2361094. 10.1080/0886022X.2024.2361094 38856016 PMC11168229

[6] Li Z Xu Z Xuan C Xu H. Association between waist triglyceride index, body mass index, dietary inflammatory index, and triglyceride- glucose index with chronic kidney disease: the 1999-2018 cohort study from NHANES. *Front Endocrinol (Lausanne).* (2024) 5:1390725. 10.3389/fendo.2024.1390725 39161393 PMC11330799

[7] Qin Y Xuan L Deng Y Wang F Liu B Wang S. Triglyceride-glucose index and mortality risk in individuals with or without chronic kidney disease: Insights from a national survey of United States adults, 1999-2018. *Nutr Metab Cardiovasc Dis.* (2024) 34:1994–2001. 10.1016/j.numecd.2024.04.003 38749783

[8] Long MT Pedley A Colantonio LD Massaro JM Hoffmann U Muntner P Development and validation of the framingham steatosis index to identify persons with hepatic steatosis. *Clin Gastroenterol Hepatol.* (2016) 14:1172.e–80.e. 10.1016/j.cgh.2016.03.034 27046482 PMC4955680

[9] Chen J Mao X Deng M Luo G. Validation of nonalcoholic fatty liver disease (NAFLD) related steatosis indices in metabolic associated fatty liver disease (MAFLD) and comparison of the diagnostic accuracy between NAFLD and MAFLD. *Eur J Gastroenterol Hepatol.* (2023) 35:394–401. 10.1097/MEG.0000000000002497 36695773 PMC9951794

[10] Han AL Lee HK. Comparison of the diagnostic performance of steatosis indices for discrimination of CT-diagnosed metabolic dysfunction-associated fatty liver disease. *Metabolites.* (2022) 12:664. 10.3390/metabo12070664 35888788 PMC9323223

[11] Cho YK Kim M Kim YJ Jung CH Lee WJ Park JY. Predictive value of the Framingham steatosis index for cardiovascular risk: a nationwide population-based cohort study. *Front Cardiovasc Med.* (2023) 18:1163052. 10.3389/fcvm.2023.1163052 37534274 PMC10391153

[12] Lai M Lai JC Allegretti AS Patidar KR Cullaro G. Investigating the association between steatotic liver disease and CKD in a nationally representative sample. *Kidney360.* (2024). 10.34067/KID.0000000569 [Epub ahead of print]. 39235870 PMC11687990

[13] Mori K Tanaka M Sato T Akiyama Y Endo K Ogawa T Metabolic dysfunction-associated steatotic liver disease (SLD) and alcohol-associated liver disease, but not SLD without metabolic dysfunction, are independently associated with new onset of chronic kidney disease during a 10-year follow-up period. *Hepatol Res.* (2024) 51:1–12. 10.1111/hepr.14097 39110552

[14] Lee HH Ro H Jung JY Chang JH Chung W Kim AJ. The fatty liver index’s association with incident chronic kidney disease in Korean middle-aged adults: a community-based cohort study. *J Clin Med.* (2024) 13:1616. 10.3390/jcm13061616 38541842 PMC10971018

[15] Li X Wang L Zhou H Xu H. Association between weight-adjusted-waist index and chronic kidney disease: a cross-sectional study. *BMC Nephrol.* (2023) 24:266. 10.1186/s12882-023-03316-w 37691097 PMC10494374

[16] Kidney Disease: Improving Global Outcomes (KDIGO) Glomerular Diseases Work Group. KDIGO 2021 clinical practice guideline for the management of glomerular diseases. *Kidney Int.* (2021) 100:S1–276. 10.1016/j.kint.2021.05.021 34556256

[17] Chambers DM Ocariz JM McGuirk MF Blount BC. Impact of cigarette smoking on volatile organic compound (VOC) blood levels in the U.S. population: NHANES 2003–2004. *Environ Int.* (2011) 37:1321–8. 10.1016/j.envint.2011.05.016 21703688

[18] Xu C Liang J Xu S Liu Q Xu J Gu A. Increased serum levels of aldehydes are associated with cardiovascular disease and cardiovascular risk factors in adults. *J Hazard Mater.* (2020) 400:123134. 10.1016/j.jhazmat.2020.123134 32569983

[19] Ochiai H Shirasawa T Yoshimoto T Nagahama S Watanabe A Sakamoto K Elevated alanine aminotransferase and low aspartate aminotransferase/alanine aminotransferase ratio are associated with chronic kidney disease among middle-aged women: a cross-sectional study. *BMC Nephrol.* (2020) 21:471. 10.1186/s12882-020-02144-6 33172399 PMC7653768

[20] Fernando B Sudeshika T Hettiarachchi T Badurdeen Z Abeysekara T Abeysundara H Evaluation of biochemical profile of chronic kidney disease of uncertain etiology in Sri Lanka. *PLoS One.* (2020) 15:e0232522. 10.1371/journal.pone.0232522 32365131 PMC7197770

[21] Qin S Wang S Wang X Wang J. Liver stiffness assessed by transient elastography as a potential indicator of chronic kidney disease in patients with nonalcoholic fatty liver disease. *J Clin Lab Anal.* (2019) 33:e22657. 10.1002/jcla.22657 30239032 PMC6818567

[22] Ardavani A Curtis F Khunti K Wilkinson TJ. The effect of pharmacist-led interventions on the management and outcomes in chronic kidney disease (CKD): a systematic review and meta-analysis protocol. *Health Sci Rep.* (2023) 6:e1064. 10.1002/hsr2.1064 36660259 PMC9840059

[23] Agidew MM Abebe EC Muche ZT Mengstie MA Mulu AT Admasu FT Evaluation of liver function biomarkers, blood pressure, and anthropometric parameters among chronic kidney disease patients: Laboratory-based cross-sectional study in Northwest Ethiopia. *Metabol Open.* (2023) 29:100254. 10.1016/j.metop.2023.100254 37681054 PMC10480547

[24] Goncharov NV Popova PI Avdonin PP Kudryavtsev IV Serebryakova MK Korf EA Markers of endothelial cells in normal and pathological conditions. *Biochem (Mosc) Suppl Ser A Membr Cell Biol.* (2020) 14:167–83. 10.1134/S1990747819030140 33072245 PMC7553370

[25] Brennan E Kantharidis P Cooper ME Godson C. Pro-resolving lipid mediators: regulators of inflammation, metabolism and kidney function. *Nat Rev Nephrol.* (2021) 17:725–39. 10.1038/s41581-021-00454-y 34282342 PMC8287849

[26] Rinella ME. Nonalcoholic fatty liver disease: a systematic review. *JAMA.* (2015) 313:2263–73. 10.1001/jama.2015.5370 26057287

[27] Li C Lin Y Luo R Chen S Wang F Zheng P Intrarenal renin-angiotensin system mediates fatty acid-induced ER stress in the kidney. *Am J Physiol Renal Physiol.* (2016) 310:F351–63. 10.1152/ajprenal.00223.2015 26672616 PMC4971807

[28] Targher G Byrne CD. Non-alcoholic fatty liver disease: an emerging driving force in chronic kidney disease. *Nat Rev Nephrol.* (2017) 13:297–310. 10.1038/nrneph.2017.16 28218263

[29] Spoto B Pisano A Zoccali C. Insulin resistance in chronic kidney disease: a systematic review. *Am J Physiol Renal Physiol.* (2016) 311:F1087–108. 10.1152/ajprenal.00340.2016 27707707

[30] Del Turco S Gaggini M Daniele G Basta G Folli F Sicari R Insulin resistance and endothelial dysfunction: a mutual relationship in cardiometabolic risk. *Curr Pharm Des.* (2013) 19:2420–31. 10.2174/1381612811319130010 23173591

[31] De La Torre A Patni N Wilson DP. Clinical management of dyslipidemia in youth with chronic kidney disease. 2024 Jun 29. In: Feingold KR Anawalt B Blackman MR Boyce A Chrousos G Corpas E editors. *Endotext (Internet).* South Dartmouth, MA: MDText.com, Inc (2000).

[32] Brar A Markell M. Impact of gender and gender disparities in patients with kidney disease. *Curr Opin Nephrol Hypertens.* (2019) 28:178–82. 10.1097/MNH.0000000000000482 30652978

[33] Pizzarelli F Basile C Aucella F Dattolo PC. Chronic kidney disease in the elderly and frail patient: perspectives with opinions and comments. *J Nephrol.* (2023) 36:1565–70. 10.1007/s40620-023-01676-y 37303023

[34] Morgenstern M Wang J Beatty N Batemarco T Sica A Greenberg H. Obstructive sleep apnea: an unexpected cause of insulin resistance and diabetes. *Endocrinol Metab Clin North Am.* (2014) 43:187–204. 10.1016/j.ecl.2013.09.002 24582098

[35] Huang X Wen S Huang Y Huang Z. Gender differences in the association between changes in the atherogenic index of plasma and cardiometabolic diseases: a cohort study. *Lipids Health Dis.* (2024) 23:135. 10.1186/s12944-024-02117-w 38715126 PMC11075304

